# Electrochemical Self-Assembled Gold Nanoparticle SERS Substrate Coupled with Diazotization for Sensitive Detection of Nitrite

**DOI:** 10.3390/ma15082809

**Published:** 2022-04-11

**Authors:** En Han, Maoni Zhang, Yingying Pan, Jianrong Cai

**Affiliations:** School of Food and Biological Engineering, Jiangsu University, Zhenjiang 212013, China; z_maoni@163.com (M.Z.); panyingying12@163.com (Y.P.)

**Keywords:** surface-enhanced Raman scattering (SERS), self-assembled AuNPs, electrochemical deposition, diazo reaction, nitrite

## Abstract

The accurate determination of nitrite in food samples is of great significance for ensuring people’s health and safety. Herein, a rapid and low-cost detection method was developed for highly sensitive and selective detection of nitrite based on a surface-enhanced Raman scattering (SERS) sensor combined with electrochemical technology and diazo reaction. In this work, a gold nanoparticle (AuNP)/indium tin oxide (ITO) chip as a superior SERS substrate was obtained by electrochemical self-assembled AuNPs on ITO with the advantages of good uniformity, high reproducibility, and long-time stability. The azo compounds generated from the diazotization-coupling reaction between nitrite, 4-aminothiophenol (4-ATP), and N-(1-naphthyl) ethylenediamine dihydrochloride (NED) in acid condition were further assembled on the surface of AuNP/ITO. The detection of nitrite was realized using a portable Raman spectrometer based on the significant SERS enhancement of azo compounds assembled on the AuNP/ITO chip. Many experimental conditions were optimized such as the time of electrochemical self-assembly and the concentration of HAuCl_4_. Under the optimal conditions, the designed SERS sensor could detect nitride in a large linear range from 1.0 × 10^−6^ to 1.0 × 10^−3^ mol L^−1^ with a low limit of detection of 0.33 μmol L^−1^. Additionally, nitrite in real samples was further analyzed with a recovery of 95.1−109.7%. Therefore, the proposed SERS method has shown potential application in the detection of nitrite in complex food samples.

## 1. Introduction

Nitrite, as a common food preservative and color retention agent as well as the main component of fertilizers, exists widely in the foodstuffs and environment [1]. For example, nitrite can maintain the red-pinkish color of the meat and prevent the risk of *Clostridium botulinum* contamination in cured meat products [2]. However, when excessive nitrite is taken into the blood, it will interfere with the oxygen transport system and cause irreversible conversion of hemoglobin to methemoglobin in the human body, by which the ability of hemoglobin to exchange oxygen is severely damaged [3,4]. Nitrite can also react with secondary amines and amides to form the carcinogenic compounds of nitrosamines, which can easily cause gastric and esophagus cancer [5,6]. Because of the toxicity of nitrite, the maximum allowable content of nitrite in drinking water recommend by the Environmental Protection Agency (EPA) is 1 ppm (71.4 μM) [7]. Hence, a rapid and sensitive detection technique developed for the measurement of nitrite is of crucial significance.

To date, numerous routine methods for the detection of nitrite have been reported such as spectrophotometry [8], chemiluminescence [9,10], capillary electrophoresis [11,12], chromatography [13,14,15], fluorescence [16,17,18,19], electrochemical methods [20,21,22], and colorimetric assay [23]. Most of the traditional methods offer a reliable detection platform for nitrite, but they also suffer from shortcomings such as multiple pretreatment procedures, long operating times, expensive instrumentation, and low sensitivity and selectivity, which limit the application of these methods in the detection of nitrite in practical food samples. Therefore, a convenient, highly sensitive, and selective analytical procedure should be developed for the determination of nitrite in complicated matrices.

Surface-enhanced Raman scattering (SERS) technology has been widely used in the detection of trace amounts of substances in recent decades due to its competitive merits such as high sensitivity and rapid detection of low concentration analytes, high specificity due to rich vibration fingerprint information with narrow Raman peaks, and simplicity and practicability with portable instrument [24,25,26]. For the better application of SERS technique, an important requirement is to fabricate highly stable, reliable, and reproducible metallic nanostructures substrates, which can generate intensively localized electromagnetic fields at gaps between plasmonic nanostructures [27,28,29]. Wang et al. [30] prepared an Au@Ag nanoparticle array sandwiched between the adhesive acrylic polymer tape and polyethene terephthalate (PET) film (T/Au@Ag/PET) as a high-performance SERS chip to detect thiram on fruit peels. Noble metal-based SERS has been widely used as a powerful analytical technique in the food detection field due to the giant signal enhancement of Raman molecules on metallic surfaces [31,32]. However, some metal nanoparticle substrate-based SERS methods need to synthesize the metal nanoparticles in solution first [1,33]. While the synthesis of gold nanoparticles (AuNPs) can be performed based on classical method developed by Turkevich et al. [34], deviations from the methodology may lead to significant nanoparticle size variation and size non-homogeneity. Furthermore, during the detection process, the nanoparticles often need to be adhered on a solid surface, which easily causes non-controlled agglomeration of the nanoparticles onto substrates for SERS and often leads to decreased signal. In contrast, the SERS substrate based on electrochemical in situ self-assembled metal nanoparticles shows enhanced uniformity, good reproducibility, and stability.

In this research, a SERS-based sensing technique combined with an electrochemical in situ self-assembled method was developed to detect nitrite in foodstuffs. AuNPs were first deposited on the clean ITO glass by the electrochemical self-assembled method. This uniform, dense, and stable SERS substrate presented extremely higher reproducibility and stability. The azo compounds generated from the diazotization reaction between nitrite, 4-aminothiophenol, and N-(1-naphthyl) ethylenediamine dihydrochloride in an acid condition were further assembled on the surface of AuNP/ITO. The intensities of three newly-observed SERS peaks, which were assigned to the formed azo compounds, were directly related to the concentration of nitrite ions. The designed SERS sensor has been demonstrated to possess high sensitivity, perfect specificity, and reproducibility for the detection of nitrite. It was further successfully applied to determine nitrite in food samples without complicated sample pretreatment, which has potential applications for the detection of trace contaminants in foodstuffs.

## 2. Experimental Methods

### 2.1. Reagents and Materials

Indium tin oxide (ITO) glasses were purchased from Shenzhen Hua-nan Technology Co., Ltd., (Shenzhen, China). Tetrachloroauric(III) acid tetrahydrate (HAuCl_4_, 99.9%), sodium nitrite (NaNO_2_, ≥99.0%), N-(1-Naphthyl) ethylenediamine dihydrohloride (NED, C_12_H_14_N_2_∙2HCl, ≥97.0%), absolute ethanol, hydrochloric acid (HCl, 36–38%), and sulfuric acid (H_2_SO_4_, 98.08%) were obtained from Sinopharm Chemical Reagent Co., Ltd., (Shanghai, China). 4-aminothiophenol (4-ATP, 97%) was purchased from Macklin Reagent Co., Ltd., (Shanghai, China). All chemicals were of analytical grade and used without further purification.

### 2.2. Apparatus

SERS measurements were performed on a handheld Raman spectrometer (HRS-5A; American Ocean optics Co., Ltd., San Diego, CA, USA) equipped with a 785 nm wavelength incident laser light. The morphologies and sizes of the AuNPs/ITO were observed by a field-emission scanning electron microscopy (FEI Quanta 250 FEG; Field Electron and Ion Ltd., Hillsboro, OR, USA). The UV-Vis absorption spectra of chips were measured by UV-Vis spectrophotometer (UV-1601; Beijing Ruili Analytical Instrument Ltd., Beijing, China).

### 2.3. Preparation of AuNPs/ITO Chip

Before the electrodeposition, ITO glass was ultrasonically cleaned for 15 min in each of the following solvents: acetone, alcohol, and Milli-Q water, and then dried in N_2_. The deposition area of the ITO glass was 7 × 10 mm^2^. The area measured in a single experiment was about 100 × 200 μm^2^. The AuNPs were deposited on cleaned ITO glass using an electrochemical workstation (CHI660D; Shanghai Chenhua Instrument Co., Ltd., Shanghai, China). A conventional three-electrode system was employed with ITO glass as the work electrode, platinum wire as the auxiliary electrode, and a saturated calomel electrode as the reference electrode. The electrodeposition of AuNPs was conducted at room temperature in the aqueous electrolyte including 5 mM HAuCl_4_ and 0.5 M H_2_SO_4_ and the potentials were set to be −0.6 V for 10 min. After deposition, the AuNPs/ITO chip was washed with ultrapure water and stored in ultrapure water.

### 2.4. Determination of Nitrite by the SERS Sensor

First, 0.05 mol L^−1^ HCl solution was obtained by diluting concentrated hydrochloric acid solution with ultrapure water. Then, 0.1 mol L^−1^ 4-ATP stock solution was made by dissolving in ethanol and further diluted with HCl solution, and NED stock solution was dissolved in water under heating conditions and diluted with water. Nitrite solutions with concentrations of 1.0 × 10^−6^, 3.0 × 10^−6^, 1.0 × 10^−5^, 3.0 × 10^−5^, 1.0 × 10^−4^, 3.0 × 10^−4^ and 1.0 × 10^−3^ mol L^−1^ were prepared. Afterward, 500 μL 4-ATP (1.0 × 10^−4^ mol L^−1^) was added into a 10 mL beaker containing 1 mL nitrite solution for the diazotization reaction. Then, the diazonium salts were mixed with 400 μL of NED, and the color of the azo dyes changed to purple-red. Finally, the mixtures were transferred into a 5 mL centrifuge tube and the AuNP/ITO substrates were immersed into 1 mL target solutions for 30 min. After being picked out from the solution, the substrates were rinsed with water three times, and then blown dry with nitrogen for SERS measurement. The SERS spectra were recorded under a 785 nm laser excitation and each sample measurement was recorded as an average of five times scans. The baseline intensity was subtracted from the peak intensity of the characteristic peak of each line to obtain the required Raman signal intensity.

### 2.5. Determination of Nitrite in Real Samples

The minced ham sausage and fresh pork were dissolved in water, then heated and stirred to dissolve the nitrite. After centrifugation, the supernatant was filtered, and then spiked with desired concentrations of nitride. For the subsequent diazo reaction, 500 μL 4-ATP was first added to 1.0 mL of the above solution and followed by the addition of 400 μL NED. Then, the AuNP/ITO chip was immersed in the solution of azo compounds generated by the above diazotization–coupling reaction and finally, SERS spectra were recorded. For the spectrophotometric method, the ham sausage and fresh pork were minced, and then saturated borax solution, potassium hexacyanoferrate solution, and zinc acetate solution were added in sequence. The supernatant was filtered and 40 mL of the above filtrate was transferred into a 50 mL colorimetric tube. Then, 2 mL sulfanilic acid solution was added to the tube, followed by the addition of 1 mL NED solution. The absorbance was measured at a wavelength of 538 nm.

## 3. Results and Discussion

### 3.1. Detection Principle of Nitrite by the Designed SERS Sensor

**Scheme 1:**
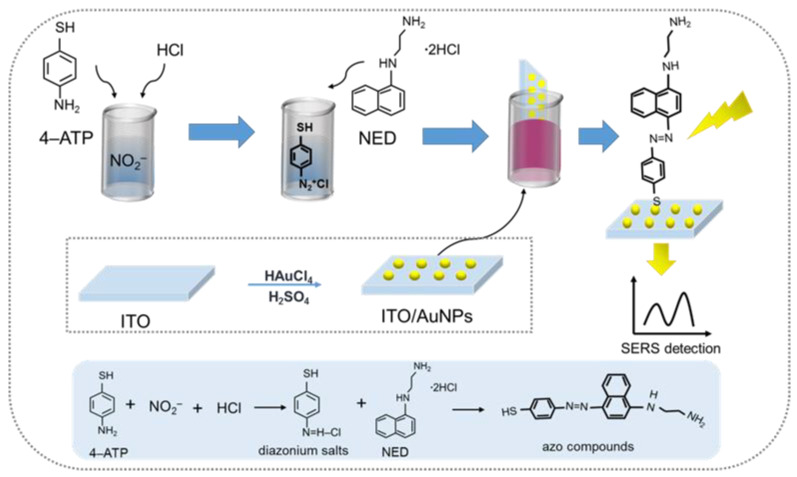
Schematic representation of the developed SERS sensing strategy for nitrite detection.

**Figure 1:**
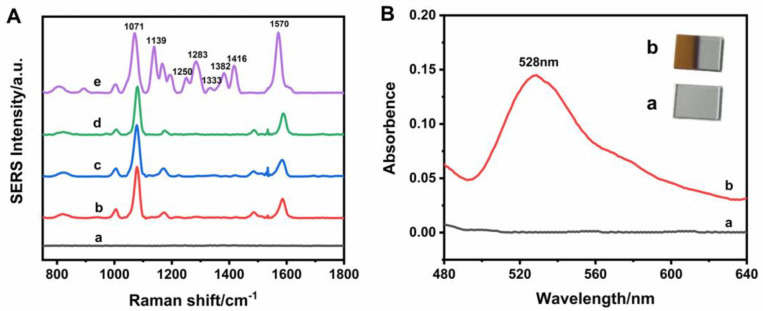
(**A**) SERS spectra of (a) blank AuNP/ITO chip, and (b) 4-ATP, (c) 4-ATP+ nitrite, (d) 4-ATP + NED, and (e) azo compounds on AuNP/ITO chip; (**B**) UV-Vis absorption spectra of (a) bare ITO and (b) AuNP/ITO film, the insert was the photos of their corresponding chips.

The SERS sensor for the quantitative detection of nitrite was fabricated by utilizing the AuNP/ITO chip as a SERS active substrate. The schematic representation of the developed SERS sensor platform is shown in Scheme 1. AuNP/ITO chip as a superior SERS substrate was obtained by electrochemical in situ self-assembled AuNPs on ITO. Then, the AuNP/ITO chip was immersed in the solution of azo compounds generated by the reaction of acid 4-ATP, NED, and nitrite to form self-assembled monolayers, which generated strong SERS signals through the SERS amplification effect. The SERS spectra for different substrates (blank AuNPs/ITO film, 4-ATP adsorbed on AuNPs/ITO film, 4-ATP and nitrite adsorbed on AuNPs/ITO film, 4-ATP and NED adsorbed on AuNPs/ITO film and azo compounds on AuNPs/ITO film) were studied. As shown in Figure 1A, the blank AuNPs on the ITO electrode surface did not present any Raman signal, which gave a “clean” Raman background, indicating that it was very suitable as the SERS substrate for further testing. As shown in cure c of Figure 1A, Raman spectrum of 4–ATP + nitrite showed a weak peak at 1430 cm^−1^, which was similar with the peak at 1432 cm^−1^ in a previous report [35]. There was no obvious change in the Raman spectrum of curve c compared with curve b because the amino group of 4–ATP was converted to diazo by nitrite in acid condition, forming unstable diazonium salts and showing weak Raman signals. NED and 4–ATP without nitrite adsorbed on the AuNP/ITO film exhibited weak SERS signals (Figure 1A, curve d). However, upon the addition of nitrite, several new peaks at the wavelengths of 1139 cm^−1^, 1283 cm^−1^, 1333 cm^−1^, 1382 cm^−1^, and 1416 cm^−1^ immediately appeared (Figure 1A, curve e), which were similar with the previous report of azo compounds generated by the diazotization reaction between nitrite, 4-ATP, and NED in an acid condition [7,25]. At this experiment, three typical peaks at 1139 cm^−1^, 1382 cm^−1^, and 1416 cm^−1^ were selected as characteristic peaks for the detection of nitrite.

### 3.2. Optimization and Characterization of the Designed AuNPs/ITO Chip

As stated in the experimental section, the AuNP/ITO chip was prepared by the electrochemical in situ self-assembled method by using HAuCl_4_ and 0.5 mol L^−1^ H_2_SO_4_ as the electrolyte. In the process of electrochemical self-assembly, the metal atoms of Au gradually assembled on the surface of ITO with the advantages of good uniformity, high reproducibility, and long-time stability. UV-Vis absorption spectra in Figure 1B show that AuNP/ITO (curve b) showed a surface plasmon resonance peak at 528 nm compared with bare ITO (curve a), which was consistent with the UV-Vis absorption peak of the AuNPs.

**Figure 2:**
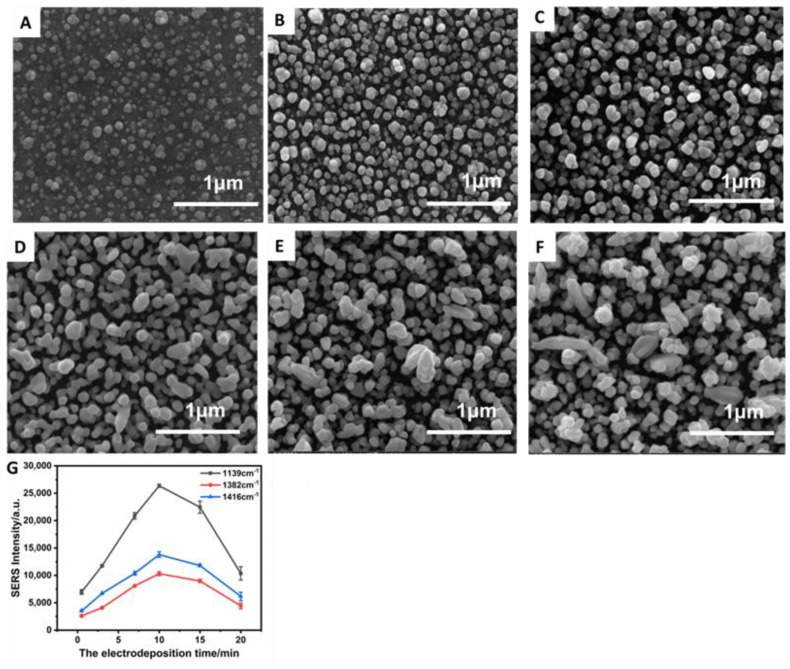
SEM images of AuNPs formed on the ITO surface for different deposition times of (**A**–**F**) (0.5, 3, 7, 10, 15, and 20 min) at −0.6 V deposition potential in 5 mM HAuCl_4_ and 0.5 M H_2_SO_4_ solution; (**G**) effect of deposition times on the SERS intensity. The error bars were derived from the relative standard deviation (RSD) of five measurements.

In order to fabricate the best SERS substrate, the electrodeposition time and concentration of HAuCl_4_ were optimized. Figure 2 shows the morphological evolution of the AuNPs in varying electrodeposition time. It was found that the electroplating time had an influence on the size of the AuNPs and the density of the coatings on the surface of ITO. When the deposition time was 0.5 min, there were lots of highly monodisperse Au nanostructures electrodeposited on the ITO electrode surface with small size and little bumps (Figure 2A). With the increase in reaction time, these gold particles grew bigger and significantly dense, and the surfaces of the AuNP/ITO chips gradually became rough. However, when the time was over 10 min, there were a few tremendous nanoparticles (Figure 2E,F). As shown in Figure 2G, the SERS signal first increased as the time of electro-deposition increased from 0.5 to 10 min, followed by a decrease in the range of 10 to 20 min. These extremely large nanoparticle structures could not stick to the ITO substrate firmly, causing the decline in the SERS peaks (Figure 2G). Consequently, the well-defined AuNP/ITO chips were obtained when the deposition time reached 10 min.

**Figure 3:**
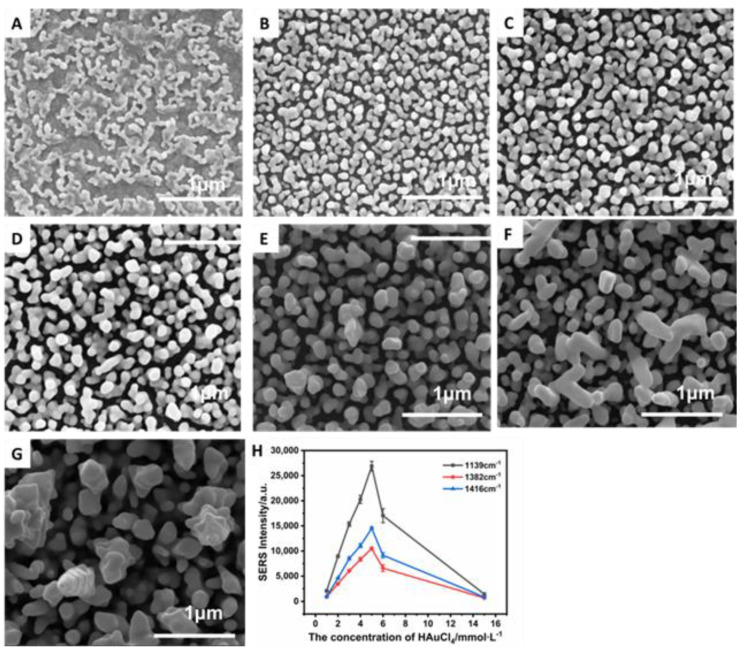
SEM images of AuNPs electrodeposited on the ITO surface using HAuCl_4_ in the concentrations of (**A**−**G**) (1, 2, 3, 4, 5, 6, and 15 mmol L**^−^**^1^) at −0.6 V deposition potential for 10 min; (**H**) effect of concentrations of HAuCl_4_ on the SERS intensity. The error bars were derived from the RSD of five measurements.

The concentration of HAuCl_4_ was also investigated to control the synthesis of the gold nanostructures on the ITO chip. As shown in Figure 3, the thickness of the gold nanoparticles increased and the size of the gold nanostructures grew bigger accordingly, with the concentration of HAuCl_4_ increasing from 1 to 15 mmol L^−1^. Obviously, the uniform and dense AuNP/ITO chips were obtained when the concentration of HAuCl_4_ was 5 mmol L^−1^. As shown in Figure 3E, the gold nanoparticles grew close to each other, this nanostructure of gold film was supposed to be the highly sensitive SERS substrate based on the electromagnetic enhancement mechanism. If HAuCl_4_ concentration was over 5 mM, there was abnormal grain growth phenomenon of AuNPs (Figure 3F,G). As shown in Figure 3H, The SERS signal increased greatly as the concentration of HAuCl_4_ changed from 1 to 5 mmol L^−1^, followed by a remarkable decrease in the concentration range of 5 to 15 mmol L^−1^. These gold nanoparticles got thicker, and the nanogap increased when the concentration increased from 5 to 15 mmol L^−1^, thereby the SERS intensity decreased sharply. Therefore, in our experiment, 5 mmol L^−1^ was chosen as the optimal concentration of HAuCl_4_ for the construction of the AuNPs/ITO chip.

### 3.3. Optimization of Experimental Conditions for the Detection of Nitrite

**Figure 4:**
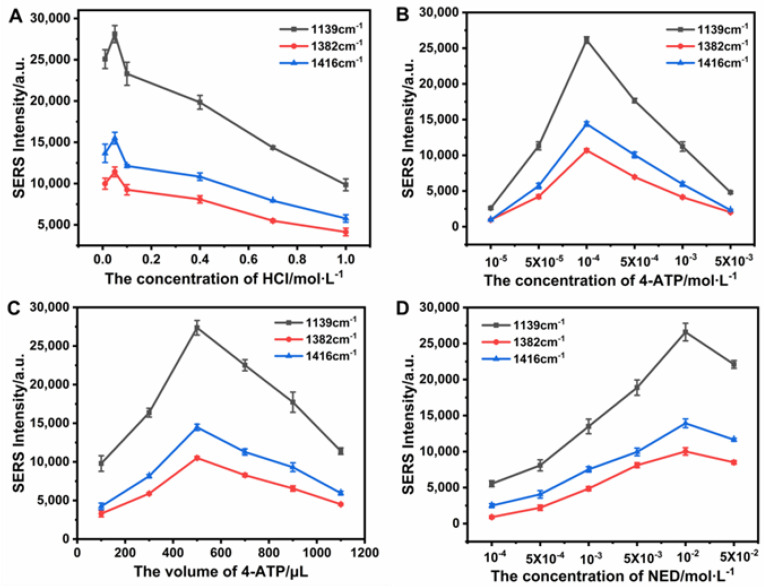
Optimization of (**A**) the concentration of HCl, (**B**) the concentration of 4-ATP, (**C**) the volume of 4-ATP, and (**D**) the concentration of NED. The error bars were derived from the RSD of five measurements.

To obtain the optimal analytical performance of the SERS sensors for nitrite detection, the concentrations of HCl, 4-ATP, and NED were investigated. First, HCl plays a decisive role in diazo reaction, thereby the concentration of HCl was optimized. As shown in Figure 4A, the best detection effect of nitrite was achieved when the concentration of HCl was 0.05 mol L^−1^. Next, the concentration and volume of 4-ATP were optimized. As shown in Figure 4B, the SERS signal first increased as the concentration of 4-ATP increased from 10^−5^ to 10^−4^ mol L^−1^, followed by decreasing in the range of 10^−4^ to 5 × 10^−3^ mol L^−1^ due to the chemical reaction kinetics. Moreover, SERS signal reached the maximum when the volume of 4-ATP increased to 500 μL (Figure 4C). Therefore, the optimal concentration and volume of 4-ATP were selected at 1 μM and 500 μL, respectively. Additionally, the effect of concentration of NED on the SERS sensor response was investigated in the range of 1.0 × 10^−4^ to 5.0 × 10^−2^ mol L^−1^. As shown in Figure 4D, the SERS signal increased greatly as the concentration of NED changed from 1.0 × 10^−4^ to 1.0 × 10^−2^ mol L^−1^, followed by a remarkable decrease in the concentration range of 1.0 × 10^−2^ to 5.0 × 10^−2^ mol L^−1^. Hence, the optimal concentration of NED was chosen at 1.0 × 10^−2^ mol L^−1^.

### 3.4. SERS Sensing of Nitrite Based on the Designed AuNPs/ITO Chip

**Figure 5:**
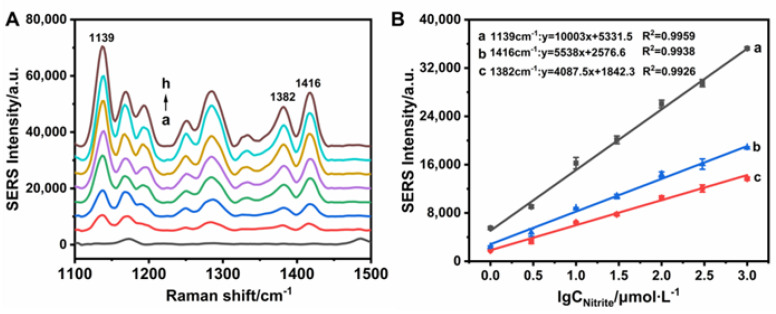
(**A**) SERS spectra of different concentrations of nitrite: (a) 0, (b) 1.0 × 10^−6^, (c) 3.0 × 10**^−^**^6^, (d) 1.0 × 10**^−^**^5^, (e) 3.0 × 10**^−^**^5^, (f) 1.0 × 10**^−^**^4^, (g) 3.0 × 10**^−^**^4^, (h) 1.0 × 10**^−^**^3^ mol L**^−^**^1^; (**B**) the correlation between SERS intensity and logarithmic value of the nitrite concentration at the bands of 1139, 1382, and 1416 cm^−1^ on AuNP/ITO chip.

Under the optimal experimental conditions, the SERS sensor exhibited sensitive response to nitrite based on AuNP/ITO chip. The results of the Raman spectroscopy are shown in Figure 5A. When the concentration of nitrite was increased, the Raman intensities of the characteristic peaks at 1139, 1382, and 1416 cm^−1^ were gradually increased. The intensity of the designed SERS sensor was proportional to the logarithmic value of the nitrite concentration ranging from 1.0 × 10^−6^ to 1.0 × 10^−3^ mol L^−1^ (Figure 5B). The limit of detection for nitrite concentration was calculated to be 0.33 × 10^−6^ mol L^−1^ at 3σ, which was lower than that of 0.23 μg mL^−1^ at the green carbon dots based fluorescence method for the detection of nitrite [17], and 2.11 μmol L^−1^ at electrochemical sensor for the detection of nitrite based on the CoPc/MWCNT electrode [36]. The detailed comparison with other methods is shown in Table 1. Clearly, the proposed electrochemical in situ self-assembled AuNP/ITO based SERS sensor for nitrite detection exhibited an excellent performance to determine trace nitrite. The results showed that the designed SERS sensor for the detection nitrite had a lower limit of detection and a wider detection range, indicating that this method would have a good application in nitrite detection.

### 3.5. Uniformity, Reproducibility, Stability, and Specificity of SERS Sensor

**Figure 6:**
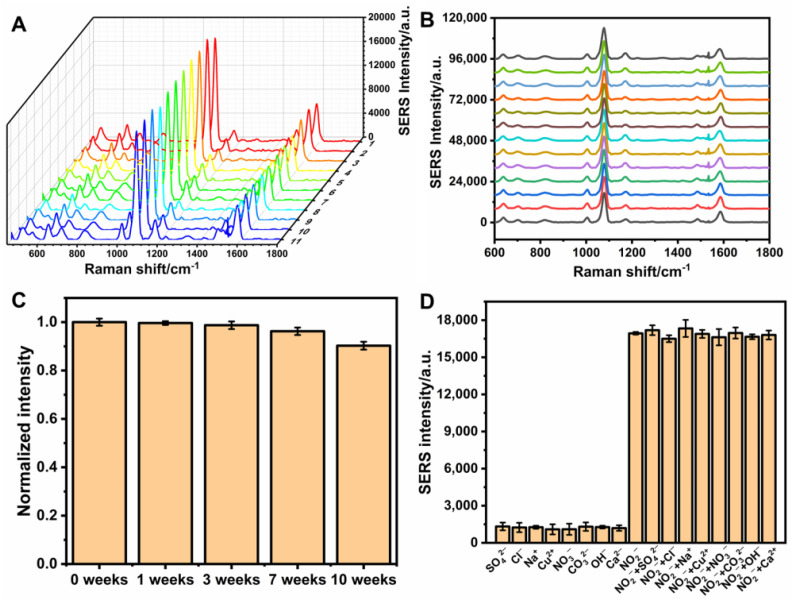
(**A**) Uniformity, (**B**) reproducibility, (**C**) stability, and (**D**) specificity of SERS sensor for the determination of nitrite.

The uniformity and reproducibility of the substrate are important factors for the SERS quantitative detection. As shown in Figure 6A, the Raman spectra were recorded by randomly selecting eleven different areas on a chip with the relative standard deviation (RSD) of 3.29%, illustrating homogeneity of SERS signals on the AuNP/ITO chip. The reproducibility of the AuNP/ITO films was investigated by the SERS spectra on 13 batches of the AuNP/ITO substrates prepared under the optimal conditions with the RSD below 4.42% (Figure 6B). In addition, the stability of Raman signal was evaluated on the basis of long-term SERS research within 12 weeks storage. As shown in Figure 6C, the decrease of SERS intensity was within 10% even when the storage time was 12 weeks. These results confirmed the excellent long-term stability of AuNP/ITO chip. Hence, the SERS substrates with good uniformity, reproducibility, and stability were successfully fabricated for the quantitative detection of nitrite.

To evaluate the specificity of the proposed SERS method, higher concentration (1.0 × 10^−3^ mol L^−1^) of interfering agents such as SO_4_^2−^, Cl^−^, Na^+^, Cu^2+^, NO^3−^, CO_3_^2−^, OH^−^, and Ca^2+^ were introduced in the nitrite sensing system under the optimum experimental conditions. The concentration of nitrite used in the interference studies was 1.0 × 10^−5^ mol L^−1^. The results of the specificity analysis are shown in Figure 6D. The observation demonstrated that the designed SERS sensor could be used to detect nitrite in the presence of other potentially interfering ions. Additionally, regeneration experiment of the self-assembled AuNP/ITO substrate by the hydrogen peroxide to cleave the gold-sulfur bond was conducted. After regeneration, the Raman signal of the designed SERS sensor could still attain 94.3% of the original response.

### 3.6. Analysis of Nitrite in Real Samples by the SERS Sensor

To further investigate the reliability and application potential for the actual sample detection of the AuNP/ITO film-based SERS sensor, the standard spiking method [3] was applied for nitrite determination using three different samples including ham sausage, fresh pork, and tap water. As shown in Table 2, the recoveries of standard additions for nitrite in the spiked samples were in the range of 95.1−109.7% with the RSD of 1.33−8.24%. The above results demonstrated that the developed SERS sensor was provided with high accuracy and satisfactory application potential, and it could be applied for the detection of nitrite in the field of food monitoring.

## 4. Conclusions

A novel SERS sensing strategy based on electrochemical self-assembled AuNP/ITO chip for rapid and sensitive determination of nitrite has been successfully designed and demonstrated. AuNPs are first deposited on the ITO glass by the electrochemical in situ self-assembled method to obtain the SERS substrate of AuNP/ITO, which presents good reproducibility and stability. The azo compounds produced by the diazotization reaction between nitrite, 4-ATP, and NED in an acid condition are then assembled on the surface of the AuNP/ITO chip. The detection of nitrite was realized based on the significant SERS enhancement of azo compounds assembled on the AuNP/ITO chip. SEM and UV-Vis were further used to characterize the self-assembled AuNP/ITO chip, and many experimental conditions were optimized such as the time of electrochemical self-assembly, the concentration of HAuCl_4_, and so on. The designed SERS sensor could detect nitride in a large linear range from 1.0 × 10^−6^ to 1.0 × 10^−3^ mol L^−1^ with a low limit of detection of 0.33 μmol L^−1^. This facile SERS sensor for the detection of nitrite has been demonstrated to possess high sensitivity, perfect specificity, and reproducibility. In addition, the designed SERS sensor was successfully applied to determine nitrite in food samples without complicated sample pretreatment, and thus could potentially become a promising technique for the assay of other trace contaminants in foodstuffs.

## Figures and Tables

**Table 1:** Comparison of the developed SERS method with the reported literatures for the detection of nitrite.

Technique	Nano Substrate	Linear Range	LOD	Ref.
SERS	Colloidal AuNPs	0.5–17 μg/mL	0.21 μg/mL	[1]
Electrochemistry	PMel ^1^/GCE	10–400 μM	1.86 μM	[6]
Fluorescence	Green carbon dots	0.4–20 μg/mL	0.23 μg/mL	[17]
Electrochemistry	AuNPs/MoS_2_	5.0–2.78 × 10^4^ μM	1.67 μM	[22]
Electrochemistry	CoPc ^2^/MWCNTs/GCE	10–1.05 × 10^6^ μM	2.11 μM	[36]
SERS	in-situ assembled AuNPs/ITO	1.0–1.0 × 10^3^ μM	0.33 μM	This work

^1^ PMel: Poly melamine; ^2^ CoPc: cobalt (II) phthalocyanine.

**Table 2:** The result of nitrite determination in real samples by developed SERS sensor.

Samples	Nitrite Added (μmol∙L^−1^)	Nitrite Found (μmol∙L^−1^)	Spectrophotometry (μmol∙L^−1^)	Recovery (%)	RSD (%)
Ham sausage	0	0.728	0.742	-	4.49
	1	1.726	1.712	99.8	2.67
	10	11.615	11.138	108.9	1.38
Fresh pork	0	0.324	0.395	-	8.24
	1	1.334	1.435	101.0	4.51
	10	11.290	11.000	109.7	1.59
Tap water	0	0	0	-	-
	1	0.951	1.088	95.1	1.33
	10	10.412	10.445	104.1	1.50

## References

[1] Wang P.X., Sun Y., Li X., Shan J.R., Xu Y., Li G.L. (2021). One-step chemical reaction triggered surface enhanced Raman scattering signal conversion strategy for highly sensitive detection of nitrite. Vib. Spectrosc..

[2] Ferysiuk K., Wójciak K.M. (2019). Reduction of Nitrite in Meat Products through the Application of Various Plant-Based Ingredients. Antioxidants.

[3] Wang Y.H., Zeng Z.X., Qiao J.Y., Dong S.Q., Liang Q., Shao S.J. (2021). Ultrasensitive determination of nitrite based on electrochemical platform of AuNPs deposited on PDDA-modified MXene nanosheets. Talanta.

[4] Yang M., Yan Y.J., Shi H.X., Liu E.Z., Hu X.Y., Zhang X., Fan J. (2020). A novel fluorescent sensors for sensitive detection of nitrite ions. Mater. Chem. Phys..

[5] Sepahvand M., Ghasemi F., Hosseini H.M.S. (2021). Plasmonic nanoparticles for colorimetric detection of nitrite and nitrate. Food Chem. Toxicol..

[6] Wu S.S., Yin Z.Z., Chen X.H., Wang X.Q., Wu D.T., Kong Y. (2020). Electropolymerized melamine for simultaneous determination of nitrite and tartrazine. Food Chem..

[7] Chen J.H., Pang S., He L.L., Nugen S.R. (2016). Highly sensitive and selective detection of nitrite ions using Fe_3_O_4_@SiO_2_/Au magnetic nanoparticles by surface-enhanced Raman spectroscopy. Biosens. Bioelectron..

[8] Ensafi A.A., Amini M. (2010). A highly selective optical sensor for catalytic determination of ultra-trace amounts of nitrite in water and foods based on brilliant cresyl blue as a sensing reagent. Sens. Actuat. B Chem..

[9] Li J.G., Li Q.Q., Lu C., Zhao L.X. (2011). Determination of nitrite in tap waters based on fluorosurfactant-capped gold nanoparticles-enhanced chemiluminescence from carbonate and peroxynitrous Acid. Analyst.

[10] Pelletier M.M., Kleinbongard P., Ringwood L., Hito R., Hunter C.J., Schechter A.N., Gladwin M.T., Dejam A. (2006). The measurement of blood and plasma nitrite by chemiluminescence: Pitfalls and solutions. Free Radic. Biol. Med..

[11] Della Betta F., Vitali L., Fett R., Costa A.C.O. (2014). Development and validation of a sub-minute capillary zone electrophoresis method for determination of nitrate and nitrite in baby foods. Talanta.

[12] Wang X., Adams E., Schepdael A.V. (2012). A fast and sensitive method for the determination of nitrite in human plasma by capillary electrophoresis with fluorescence detection. Talanta.

[13] Chiesa L., Arioli F., Pavlovic R., Villa R., Panseri S. (2019). Detection of nitrate and nitrite in different seafood. Food Chem..

[14] Luckovitch N., Pagliano E. (2019). A reference isotope dilution headspace GC/MS method for the determination of nitrite and nitrate in meat samples. Int. J. Food Sci. Technol..

[15] Coviello D., Pascale R., Ciriello R., Salvi A.M., Guerrieri A., Contursi M., Scrano L., Bufo S.A., Cataldi T.R.I., Bianco G. (2020). Validation of an Analytical Method for Nitrite and Nitrate Determination in Meat Foods for Infants by Ion Chromatography with Conductivity Detection. Foods.

[16] Georgescu-State R., van Staden J.F., Popescu-Mandoc L.R. (2018). Fluorimetric determination of nitrite in water using a novel fluorescent dye. Microchem. J..

[17] Yue X.Y., Zhou Z.J., Wu Y.M., Jie M.S., Li Y., Guo H.B., Bai Y.H. (2020). A green carbon dots-based fluorescent sensor for selective and visual detection of nitrite triggered by the nitrite-thiol reaction. New J. Chem..

[18] Li B.L., Li Y.S., Wu Y., Gao X.F. (2019). Fluorescence quenching capillary analysis for determining trace-level nitrite in food based on the citric acid/ethylenediamine nanodots/nitrite reaction. Food Chem..

[19] Li W.T., Shi Y.Q., Hu X.T., Li Z.H., Huang X.W., Holmes M., Gong Y.Y., Shi J.Y., Zou X.B. (2019). Visual detection of nitrite in sausage based on a ratiometric fluorescent system. Food Control.

[20] Ge Y., Jamal R., Zhang R.Y., Zhang W.L., Yu Z.N., Yan Y.Q., Liu Y.C., Abdiryim T. (2020). Electrochemical synthesis of multilayered PEDOT/PEDOT-SH/Au nanocomposites for electrochemical sensing of nitrite. Microchim. Acta.

[21] Wang X., Li M.J., Yang S., Shan J.J. (2020). A novel electrochemical sensor based on TiO_2_-Ti_3_C_2_T_X_/CTAB/chitosan composite for the detection of nitrite. Electrochim. Acta.

[22] Zhang S., Tang Y.P., Chen Y.Y., Zheng J.B. (2019). Synthesis of gold nanoparticles coated on flower-like MoS_2_ microsphere and their application for electrochemical nitrite sensing. J. Electroanal. Chem..

[23] Trofimchuk E., Hu Y.X., Nilghaz A., Hua M.Z., Sun S., Lu X.N. (2020). Development of paper-based microfluidic device for the determination of nitrite in meat. Food Chem..

[24] Jiang L., Wang L., Zhan D.S., Jiang W.R., Fodjo E.K., Hafez M.E., Zhang Y.M., Zhao H., Qian R.C., Li D.W. (2021). Electrochemically renewable SERS sensor: A new platform for the detection of metabolites involved in peroxide production. Biosens. Bioelectron..

[25] Pang S., Yang T.X., He L.L. (2016). Review of surface enhanced Raman spectroscopic (SERS) detection of synthetic chemical pesticides. TrAC Trend. Anal. Chem..

[26] He G.Y., Han X.Y., Cao S.Y., Cui K.M., Tian Q.H., Zhang J.H. (2022). Long Spiky Au-Ag Nanostar Based Fiber Probe for Surface Enhanced Raman Spectroscopy. Materials.

[27] Tong Q., Wang W.J., Fan Y.N., Dong L. (2018). Recent progressive preparations and applications of silver-based SERS substrates. TrAC Trend. Anal. Chem..

[28] Perumal J., Wang Y.S., Attia A.B.E., Dinish U.S., Olivo M.C. (2021). Towards a point-of-care SERS sensor for biomedical and agri-food analysis applications: A review of recent advancements. Nanoscale.

[29] Huang Z.C., Zhang A., Zhang Q., Cui D.X. (2019). Nanomaterials-based SERS Sensing Technology for Biomedical Application. J. Mater. Chem. B.

[30] Wang K.Q., Sun D.W., Pu H.B., Wei Q.Y. (2021). Polymer multilayers enabled stable and flexible Au@Ag nanoparticle array for nondestructive SERS detection of pesticide residues. Talanta.

[31] Luo Y.H., Wen G.Q., Dong J.C., Liu Q.Y., Liang A.H., Jiang Z.L. (2014). SERS detection of trace nitrite ion in aqueous solution based on the nitrosation reaction of rhodamine 6G molecular probe. Sens. Actuat. B Chem..

[32] Zheng P., Kasani S., Shi X.F., Boryczka A.E., Yang F., Tang H.B., Li M., Zheng W.H., Elswick D.E., Wu N.Q. (2018). Detection of nitrite with a surface-enhanced Raman scattering sensor based on silver nanopyramid array. Anal. Chim. Acta.

[33] Lu Y.D., Lu D.C., You R.Y., Liu J.L., Huang L.Q., Su J.Q., Feng S.Y. (2018). Diazotization-Coupling Reaction-Based Determination of Tyrosine in Urine Using Ag Nanocubes by Surface-Enhanced Raman Spectroscopy. Nanomaterials.

[34] Turkevich J., Stevenson P.C., Hillier J. (1951). A study of the nucleation and growth processes in the synthesis of colloidal gold. Discuss. Faraday Soc..

[35] Ahmad W., Wang J.J., Wu L.H., Zhu J.J., He P.H., Ouyang Q., Chen Q.S. (2020). Design of Physicochemical Factors for Regulating the Retention Mechanism of 4-aminothiophenol in Surface-Enhanced Raman Scattering toward Nitrite Sensing. J. Phys. Chem. C.

[36] Lu S., Hummel M., Kang S., Gu Z. (2020). Selective voltammetric determination of nitrite using cobalt phthalocyanine modified on multiwalled carbon nanotubes. J. Electrochem. Soc..

